# Leucine Rich α-2 Glycoprotein: A Novel Neutrophil Granule Protein and Modulator of Myelopoiesis

**DOI:** 10.1371/journal.pone.0170261

**Published:** 2017-01-12

**Authors:** Lawrence J. Druhan, Amanda Lance, Shimena Li, Andrea E. Price, Jacob T. Emerson, Sarah A. Baxter, Jonathan M. Gerber, Belinda R. Avalos

**Affiliations:** 1 The Department of Hematologic Oncology, The Levine Cancer Institute, Carolinas HealthCare System, Charlotte, North Carolina, United States of America; 2 The University of North Carolina School of Medicine, Chapel Hill, North Carolina, United States of America; Wayne State University, UNITED STATES

## Abstract

Leucine-rich α2 glycoprotein (LRG1), a serum protein produced by hepatocytes, has been implicated in angiogenesis and tumor promotion. Our laboratory previously reported the expression of LRG1 in murine myeloid cell lines undergoing neutrophilic granulocyte differentiation. However, the presence of LRG1 in primary human neutrophils and a role for LRG1 in regulation of hematopoiesis have not been previously described. Here we show that LRG1 is packaged into the granule compartment of human neutrophils and secreted upon neutrophil activation to modulate the microenvironment. Using immunofluorescence microscopy and direct biochemical measurements, we demonstrate that LRG1 is present in the peroxidase-negative granules of human neutrophils. Exocytosis assays indicate that LRG1 is differentially glycosylated in neutrophils, and co-released with the secondary granule protein lactoferrin. Like LRG1 purified from human serum, LRG1 secreted from activated neutrophils also binds cytochrome c. We also show that LRG1 antagonizes the inhibitory effects of TGFβ1 on colony growth of human CD34^+^ cells and myeloid progenitors. Collectively, these data invoke an additional role for neutrophils in innate immunity that has not previously been reported, and suggest a novel mechanism whereby neutrophils may modulate the microenvironment via extracellular release of LRG1.

## Introduction

Neutrophils are traditionally viewed as the “first responders” of the innate immune system, owing to their intrinsic capacity to eliminate pathogenic organisms. An array of proteins reside within neutrophils that are packaged into cytoplasmic granules and released at sites of infection to help eliminate foreign pathogens. Four distinct subsets of neutrophil granules have been identified: primary, secondary, and tertiary granules, and secretory vesicles. Protein packaging into the various granule subsets equips neutrophils for rapid and differential release of high concentrations of proteins following neutrophil activation. Tertiary granules are released during neutrophil extravascularization, while primary and secondary granules are exocytosed upon entry of neutrophils into the extravascular space. Although a predominant antimicrobicidal function has been ascribed to neutrophil granule proteins, recent evidence suggests that neutrophil granule proteins can also modulate the microenvironment.

Leucine rich α-2 glycoprotein (LRG1), the founding member of the leucine rich repeat (LRR) family of proteins, was initially purified from the serum of healthy individuals and found to be present at concentrations ranging from 10–50 ug/mL [1, 2]. Subsequent studies have shown that LRG1 expression increases in hepatocytes in response to mediators of the acute-phase response, and serum LRG1 levels are increased in patients with bacterial infections [3, 4]. Increased concentrations of LRG1 have also been observed in a variety of neoplastic and inflammatory disorders, prompting a number of investigators to suggest that LRG1 is a biomarker of these diseases [5–8]. Additionally, our group previously reported that transcription of LRG1 is upregulated in myeloid cells during granulopoiesis and ectopic overexpression of LRG1 accelerates granulopoiesis *in vitro*, suggesting a role for LRG1 in myeloid cell maturation [9]. LRG1 has also been reported to bind cytochrome c, the physiologic relevance of which remains unclear [10, 11].

Cytochrome c is a mitochondrial electron transfer protein involved in oxidative phosphorylation in a key apoptotic cell death pathway. Release of cytochrome c from the mitochondria to the cytosol is initiated by various stimuli and leads to formation of the apoptosome, activation of effector caspase enzymes, and subsequent cell death [12]. Cytochrome c has also recently been reported to exert effects extracellularly. Extracellular cytochrome c is implicated in the initiation of arthritis via the NF-kB pathway, suggesting it can function as a pro-inflammatory molecule [13]. In addition, treatment of lymphocytes with extracellular cytochrome c was reported to induce apoptosis, which was mitigated by the presence of LRG1 [11].

The pleiotropic activities of LRG1 appear to be mediated by its interactions with other proteins through the formation of protein:protein complexes. Like many members of the LRR family, LRG1 has multiple binding partners. In addition to cytochrome c, LRG1 also binds TGFβ1 [14]. Formation of protein:protein complexes between LRG1 and the TGFβ-accessory receptors was recently reported to promote angiogenesis through a process that was absolutely dependent on the presence of TGFβ1. [15].

Based on our prior studies using murine myeloid cell lines, we were interested in exploring the tantalizing possibility that LRG1 is packaged into cytoplasmic granules in primary human neutrophils and released extracellularly following neutrophil activation to modulate the microenvironment. Here we show that LRG1 is synthesized in human neutrophils during neutrophil granulocyte differentiation, packaged into peroxidase-negative granules, and exocytosed following neutrophil activation. Moreover, we demonstrate that neutrophil-derived LRG1 binds to cytochrome c in a manner similar to LRG1 purified from serum. Additionally, we show that LRG1 attenuates the anti-proliferative effects of TGFβ on hematopoiesis. Collectively, our data suggest a potential role for neutrophils in modulating the microenvironment through the release of LRG1 from activated neutrophils.

## Materials and Methods

### Isolation of peripheral blood neutrophils

Peripheral blood was obtained from healthy volunteer donors through a protocol approved by the Chesapeake IRB and informed written consent obtained in accordance with the Declaration of Helsinki. For isolation of peripheral blood neutrophils, blood was collected in ACD-containing tubes, and the neutrophils isolated using a combination of dextran sulfate sedimentation and density gradient centrifugation (Histopaque 1.077), followed by hypotonic lysis, as previously described [16]. Cell counts and viability (>99%) were assessed using the Muse Cell Analyzer (Millipore) and the companion count and viability kit (MCH100102). The purity of the isolated monocytes (>97%) was determined by morphologic examination of cells cytospun onto slides and stained with Wright-Giesma.

### ELISA and immunoblotting

Samples were electrophoresed on 4–20% sodium dodecyl sulfate polyacrylamide (SDS-PAGE) gels under reducing conditions and transferred to nitrocellulose using standard protocols. The membranes were incubated with the indicated primary antibodies overnight at 4°C, then incubated with the relevant horseradish peroxidase (HRP)-labeled secondary antibody for 2 hours at room temperature (RT). Immunoreactive proteins were detected using enhanced chemiluminescence (BioRad), and visualized on a ChemiDoc-It^2^ Imager (Ultraviolet Laboratory Products). The antibodies used were goat anti-lactoferrin (LF) (Santa Cruz sc14431), goat anti-myeloperoxidase (MPO) (Santa Cruz sc34159), goat anti-matrix metalloproteinase 9 (MMP9) (Santa Cruz sc13520), and rabbit anti-LRG1 (Abcam ab170953). The ELISA kits used were human LRG1 ELISA (IBL 27769), human MPO ELISA (IBL IB39547), human LF ELISA (Abcam ab108882), and human MMP9 ELISA (Abcam ab100610). For the LRG1 ELISA, samples were heated to 95°C and then cooled to RT prior to measurement.

### Neutrophil exocytosis

Isolated human neutrophils were resuspended at 1 x 10^6^ cells per mL in Hanks Balanced Salt Solution (HBSS) containing calcium and magnesium (Lonza 10-527F), preincubated at 37°C for 5 minutes, after which calcium ionophore (1uM), phorbol myristate acetate (PMA) (25ng/mL), f-methyl-phenylalanine (fMLP) (100nM), or vehicle (control) was added, and the cells further incubated at 37°C for 20 minutes, then transferred to ice. The cells were pelleted by centrifugation and the supernatants collected, fast frozen in liquid nitrogen, and stored at -80°C until analysis. Western blot and ELISA analyses were performed to measure the amount of the individual granule proteins released.

### Subcellular fractionation

Neutrophil subcellular fractions were generated as previously described with slight modifications [17]. Isolated neutrophils were incubated in 5 mM Pefabloc in HBSS without calcium and magnesium for 5 minutes, spun down, and resuspended in 10mL Disruption Buffer (calcium and magnesium-free HBSS with 1mM ATP (Na)_2_ and 100ul of 100mM Pefabloc). Neutrophil suspensions were then disrupted by nitrogen cavitation in a cell disruption vessel (Parr Instruments Co. Cat. no 4635) by pressurizing the cell suspension to 350–375 psi for 5 minutes, and collecting the cavitates drop-wise into a tube containing ethylene glycol tetra-acetic acid (EGTA) (150ul of 100mM). The cavitates were centrifuged at 400g for 15 min and the postnuclear supernatants layered onto a three layer percoll gradient (1.05, 1.09, and 1.12 g/mL). Each gradient was made using a 1.13 g/mL Percoll solution diluted in Disruption Buffer containing 10 mM ATP[Na]2 and 0.5 mM PMSF. The gradient was centrifuged at 32,000g for 55 min at 4°C and 1 mL fractions were collected from the bottom of the tube.

### Immunofluorescence microscopy

Isolated neutrophils were cytospun onto slides, fixed in methanol for 10 minutes, rinsed with PBS, then incubated in PBS with 0.25% Triton X-100 and 1% fetal bovine serum (FBS) for 1hr at RT. Primary antibodies were diluted in blocking solution, added to the slide, and incubated overnight at 4°C. The following day, the slides were washed twice with PBS, and incubated with fluorescently labeled secondary antibodies for 1.5 hr at RT in the dark. The slides were then rinsed twice with PBS and coverslips affixed to the slides using Prolong Gold with 4',6-diamidino-2-phenylindole (DAPI) (ThermoFisher Sci. P36931). The slides were then analyzed using a Zeiss LSM 710 confocal system and a 63x oil objective.

### Protein identification by LC/MS/MS

Commercially available LRG1 purified from human serum (MyBioSource) and LRG1 isolated from neutrophil granule releasates were loaded onto a 4–20% SDS-PAGE gel under reducing conditions and silver stained using Pierce Silver Stain for Mass Spectrometry (ThermoFisher Scientific). Protein bands corresponding to the molecular weight of LRG1 were excised, trypsin digested, and analyzed on a Thermo Scientific LTQ-XL mass spectrometer. The MS raw data was submitted to Sequest and searched against the human UniProtKB database (release-2014_10).

### Deglycosylation studies

Lysates were prepared from isolated neutrophils by resuspending the cells in Radioimmunoprecipitation assay buffer (RIPA) containing a cocktail of protease inhibitors, and subjecting the cell suspensions to pulse sonication. Neutrophil granule releasates were prepared by exposing isolated neutrophils to calcium ionophore (2 uM) after which the released material was concentrated using a Millipore Amicon Ultra 3K molecular weight cut off filter. Commercially available LRG1 isolated from human serum (My BioSource), neutrophil lysates and neutrophil granule releasates were deglycosylated using a commercially available protein deglycosylation kit (Promega cat. no. V2931) according to the manufacturer’s instructions. Samples were electrophoresed on 4–20% SDS-PAGE gels under reducing conditions, transferred to nitrocellulose, and incubated with an antibody to human LRG1 (Abcam ab178698).

### Cytochrome c affinity chromatography

Cytochrome c from equine heart tissue (Sigma C2506) was solubilized in PBS at a concentration of 5 mg/mL. This solution was used as the coupling ligand for generation of an affinity column using a 1 mL HiTrap N-hydroxysuccinimide (NHS)-activated HP sepharose prepacked column from GE Healthcare (17-0716-01), following the manufactures’ instructions. The resultant cytochrome c affinity column was equilibrated in PBS, and total releasate from neutrophils (25 million) stimulated for 20 minutes with calcium ionophore (2 uM) was loaded onto the column. The column was washed with two column volumes of PBS. LRG1 was eluted with an acetate buffer (pH 4) in 1 mL fractions.

### Cell culture, viability, and differentiation assays

HL-60 cells (ATCC CCL240) were maintained in Roswell Park Memorial Institute medium (RPMI) 1640 (Life Technologies 21870092), 10% FBS, 2 mM glutamine, and Penicillin/Streptomycin (Invitrogen 15140–122). Differentiation was induced by the addition of all trans-retinoic acid (ATRA) (Sigma R2625) at a final concentration of 1 uM. At the indicated time points, cells were harvested, lysed in RIPA buffer containing protease inhibitors, and analyzed for the presence of LRG1. For HL-60 proliferation experiments, 50,000 cells per well were seeded in serum-free RPMI supplemented with insulin/transferrin/selenite supplement (ITS) (Sigma I3146) into 96 well plates. The cells were incubated with the indicated treatments for 48 hours, and cell proliferation measured using an XTT assay kit (ATCC 30-1011K).

### Methocult colony assays

CD34+ cells were isolated from human bone marrow samples from healthy volunteer donors through an IRB-approved protocol using Miltenyi Biotec human magnetic selection microbeads (Cat# 130-046-703), LS column (Cat# 130-042-401) and QuadroMAX magnetic separator (Cat# 130-091-051). Positively selected cells were counted and diluted in serum free UltraCulture media (Fisher Scientific, Cat# 12-725F). For each treatment condition, the indicated amount of TGF-B (Sigma, Cat#T7039) and/or LRG1 (Sino Biological, Cat#13371-HCCH) was added to a 4 mL methocult aliquot, vortexed, and 2,000 CD34+ selected cells added. For each experimental condition, a total of 1.1mL of CD34+ cells in the methocult solution was pipetted in triplicate via a 16G syringe into 35mm plates, and incubated for 14 days at 37°C in a humidified incubator containing 5% CO2. The plates were scored and counted by three independent investigators using an inverted microscope. To assess cell morphology and number, individual colonies were plucked from the methocult cultures using a micropipette, resuspended in 300 uL of HBSS, cytospun onto slides, and stained with Wright-Giesma. All colony assay experiments were performed in triplicate.

## Results

### LRG1 protein is synthesized during neutrophil granulocyte differentiation and packaged into neutrophil granules

**Fig 1:**
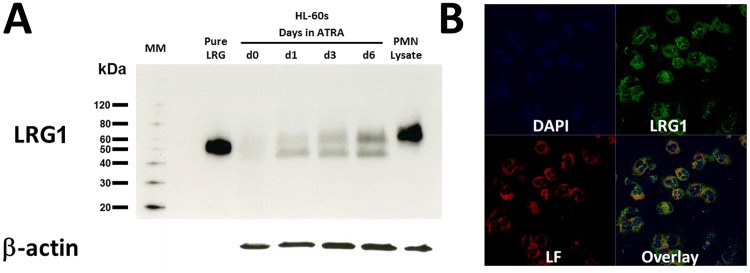
LRG1 is expressed during neutrophilic granulocyte differentiation and stored in lactoferrin (LF)-containing granules. **(A)** HL-60 cells were induced to differentiate with ATRA (1 uM) for 6 days, and soluble proteins extracted from cells harvested at days 0, 1, 3, and 6. Samples were subjected to immunoblot analysis to detect the presence of LRG1. Pure LRG1 in lane 1 is LRG1 purified from human serum included as a positive control. Proteins isolated from unstimulated human neutrophils (PMN lysate) are shown in the last lane. (**B)** Confocal immunofluorescence microscopy of purified human neutrophils incubated overnight with mouse anti-LRG1 (Abnova H00116844-M01, 1:100 dilution) and goat anti-LF (Santa Cruz sc-14431, 1:250 dilution), followed by donkey anti-mouse Alexafluor 488 (green, 1:500 dilution) and donkey anti-goat Alexafluor 594 (red, 1:2000 dilution) secondary antibodies. DAPI was used to stain nuclei (blue). LRG1 is visualized as green and LF as red. The yellow/orange color in the overlay panel is the result of green and red signals merging, indicating co-localization of LRG1 (green) with LF (red). Focal areas of non-merged green and red signals can also be seen, suggesting that LRG1 may also localize to non-LF-containing compartments. The data shown are representative of 3 independent experiments.

We previously reported that transcription of LRG1 is upregulated during neutrophilic granulocyte differentiation of murine 32Dcl3G cells [9]. To determine whether the observed increase in LRG1 transcription during neutrophil differentiation is accompanied by an increase in protein expression that is physiologically relevant in human cells, lysates from HL-60 cells induced to differentiate in response to ATRA as well as lysates from neutrophils from healthy human volunteer donors were subjected to immunoblot analysis for LRG1. As shown in Fig 1A, expression of LRG1 protein increases in HL-60 cells induced to differentiate in response to ATRA with higher expression levels observed as maturation proceeds along the neutrophil lineage. A high level of expression of LRG1 protein is readily detected in neutrophils isolated from the peripheral blood of healthy human donors (Fig 1A, last lane). Notably, LRG1 from human neutrophils exhibits a higher molecular weight (~ 60 kDa) than LRG1 purified from human serum (~ 50 kDa). To confirm that the higher molecular weight species detected in neutrophils is, indeed, LRG1, the immunoreactive band migrating at 60 kDa was excised from the SDS-PAGE gel and subjected to proteomic analysis. Using mass spectrometry (LC/MS/MS), we confirmed the identity of the band in neutrophils as human LRG1 (S1 Fig).

We next investigated the subcellular localization of LRG1 in human neutrophils. Using confocal immunofluorescence microscopy, LRG1 (green) can be seen to localize to the cytoplasmic compartment (Fig 1B, *upper right panel*) in a pattern that partially overlaps with lactoferrin (LF, red; Fig 1B, *lower left panel*). Focal areas of non-merged green and red signals evident in the overlaid images (Fig 1B, *lower right panel*) suggest that LRG1 may also localize to a non-LF-containing granule compartment.

### LRG1 protein localizes to the peroxidase-negative granule compartment of neutrophils

In order to more definitively determine the subcellular localization of LRG1 within human neutrophils, we employed a technique utilizing nitrogen cavitation followed by percoll density-gradient centrifugation. Nitrogen cavitation is a gentle method for rupturing the plasma membrane of neutrophils without disturbing the membrane surrounding neutrophil granules [18]. Centrifugation over a three step percoll density gradient can then be used to separate the various neutrophil granule compartments that can be identified by the presence of specific proteins known to reside within each granule compartment: myeloperoxidase (MPO) for primary granules, lactoferrin (LF) for secondary granules, and gelatinase or matrix metalloproteinase 9 (MMP9) for tertiary granules.

**Fig 2:**
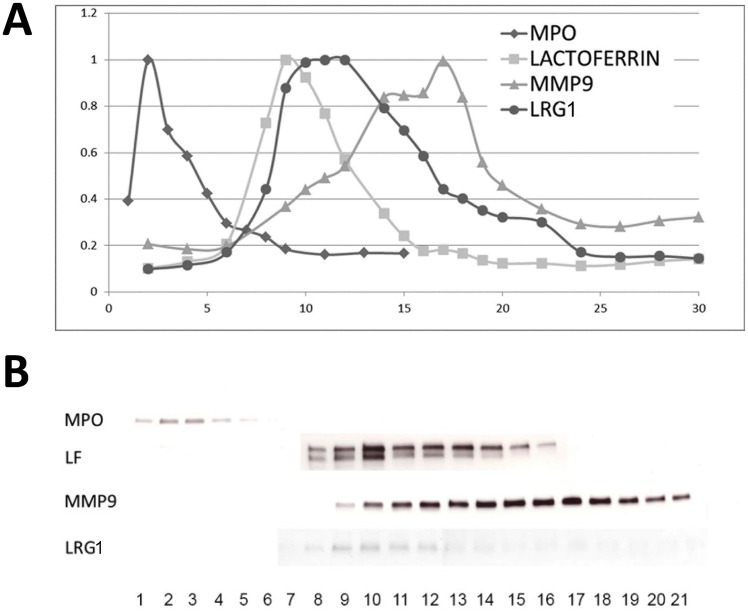
LRG1 predominantly localizes to the secondary granule compartment of neutrophils. **(A)** Neutrophils were isolated from the peripheral blood of healthy donors as described, lysed by nitrogen cavitation, and the subcellular components separated by Percoll density centrifugation. The fractions are numbered 1 through 30, with fraction 1 corresponding to the most dense fraction and fraction 30 corresponding to the least dense fraction. Fractions were analyzed by ELISA for the presence of LRG1, MPO (primary granule marker), LF (secondary granule marker), and MMP9 (tertiary granule marker), and the results plotted as a function of dentisty. **(B)** Immunoblot analyses of fractions 1 through 21 from the same experiment presented in panel A, showing peak concentrations for MPO, LF, and MMP9 in fractions 2, 10, and 17, respectively, identical to the fractions containing the highest concentration of the indicated proteins as detected by ELISA shown in panel A. The peak concentration of LRG1 is detected in fraction 10, which corresponds to the peroxidase-negative LF-containing secondary granule compartment, consitant with the ELISA data presented in Panel A. The data shown are representative of 5 independent experiments.

Aliquots from individual fractions were collected and subjected to ELISA analyses for MPO, LF, MMP9, and LRG1 (Fig 2A). The identical fractions were also analyzed by western blot (Fig 2B). Analysis of the ELISA data in Fig 2A clearly shows that LRG1 does not co-localize with MPO, but localizes with fractions corresponding to the secondary granule marker LF. Notably, the LRG1 curve is broad with a large shoulder, and indicates significant overlap with the tertiary granule marker, MMP9. Immunoblot analyses shown in Fig 2B, confirm that LRG1 co-localizes with the LF-containing secondary granules and the higher density granules containing MMP9.

### LRG1 is released by activated neutrophils

**Fig 3:**
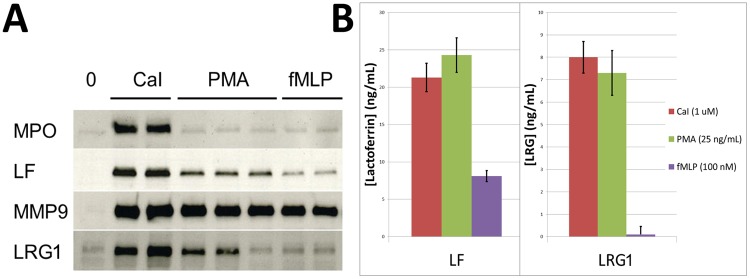
Activated neutrophils release LRG1. Human neutrophils isolated from healthy donors were pre-incubated in HBSS containing calcium and magnesium at 37°C for 5 minutes, then incubated for 20 minutes at 37°C with the indicated concentrations of calcium ionophore (CaI), PMA, fMLP, or no stimulant (0, negative control). The supernatants were collected, and immunoblot and ELISA analyses performed to detect and measure the amount of granule proteins released. **(A)** Immunoblot analysis of each condition from a representative experiment using antibodies for myeloperoxidase (MPO), lactoferrin (LF), and gelatinase (MMP9), as markers for primary, secondary, and tertiary granule release, respectively. The pattern of stimulation for release of LRG1 is most consistent with its release from LF-containing secondary granules. The data shown are representative of three independent experiments, each with a different normal donor. **(B)** ELISA analyses demonstrate release of LRG1 from neutrophils following stimulation with Cal or PMA but not with fMLP, a pattern consistent with the release of LF from secondary granules. The mean of three independent experiments is shown and error bars represent standard error (n = 3).

Neutrophil granule release can be initiated by a variety of stimulants that are capable of mobilizing specific subsets of granules [19]. Calcium ionophore is known to stimulate the release of all granule subtypes. PMA stimulates release of secondary and tertiary granules, while fMLP stimulates release of tertiary granules. To determine if LRG1 is, indeed, secreted by activated human neutrophils, and, if so, which stimulants induce exocytosis of the LRG1-containing granules, isolated neutrophils were stimulated with caIcium ionophore, PMA, and fMLP. Supernatants were harvested from neutrophils following incubation with the various stimulants, and analyzed by immunoblot analyses and ELISA. As shown in Fig 3, activated neutrophils release LRG1 in response to calcium ionophore and PMA, but not to fMLP. The release of LRG1 by stimulants known to induce release of secondary granule proteins is consistent with our localization studies showing that LRG1 co-localizes with the secondary granule protein LF.

### Neutrophil-derived LRG1 is differentially glycosylated and exhibits similar binding properties as serum LRG1

**Fig 4:**
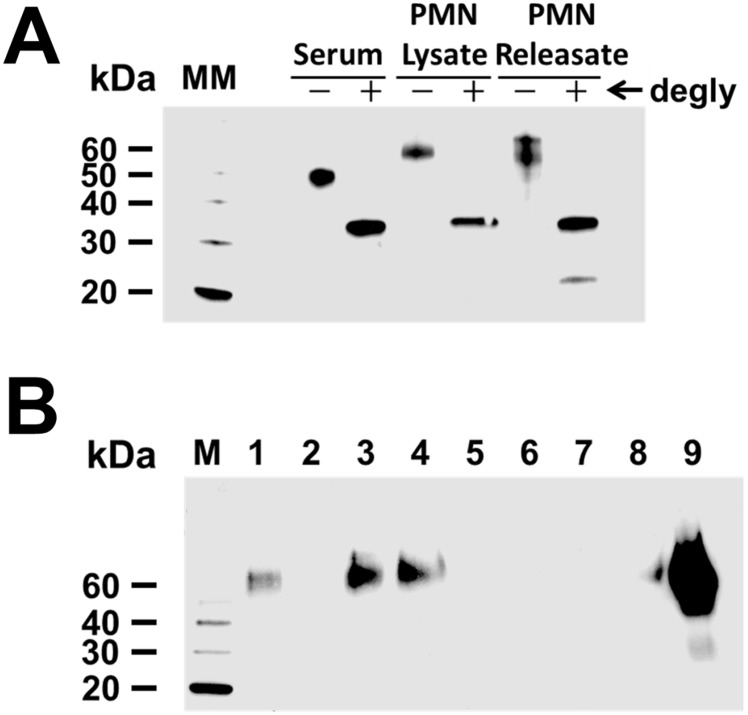
Neutrophil-derived LRG1 is differentially glycosylated but retains affinity for cytochrome c. Neutrophil (PMN) lysates from healthy volunteers were prepared as described. PMN releasates were prepared by exposing isolated neutrophils to calcium ionophore and the resultant supernatants concentrated by ultrafiltration. **(A)** LRG1 purified from human serum, PMN lysate, and PMN granule releasate were left untreated (- lanes) or subjected to deglycosylation (+ lanes) using a commercially available protein deglycosylation kit, and immunoblot analysis performed using an antibody to human LRG1. Molecular weight markers (MM) are shown on the left. Neutrophil-derived LRG1 migrates with a higher molecular weight than serum-derived LRG1 (- lanes) but with the same molecular weight following deglycosylation (+ lanes), indicating different patterns of glycosylation of the native protein forms in PMNs compared to serum. The data shown are representative of three independent experiments, each with a different normal donor. **(B)** Purified cytochrome c was bound to a NHS-activated sepharose column. Releasate from PMNs stimulated with calcium ionophore (2 uM) was loaded onto the cytochrome c column. Bound proteins were eluted with an acetate buffer (pH 4) in 1 mL fractions, and subjected to immunoblot analysis with antibody to LRG1. Lane 1 is crude PMN releasate. The column flow through and wash is shown in lane 2. Eluate fractions are shown in lanes 3–8, with LRG1 detectable in fractions 3 and 4. Lane 9 is purified LRG1. The data shown are representative of three independent experiments.

Based on previously published studies on haptoglobin [20], we hypothesized that the observed difference in molecular weight between LRG1 isolated from human serum and LRG1 isolated from human neutrophils might be due to differences in glycosylation. To determine whether this was the case, LRG1 purified from human serum, lysates from human neutrophils, and concentrated releasates from activated neutrophils was subjected to deglycosylation. As shown in Fig 4A, immunoblot analysis of the deglycosylated samples shows that both serum and neutrophil-derived LRG1 (lysates and releasates) migrate with the identical molecular weight, indicating that the difference in apparent molecular weight of neutrophil and serum derived LRG1 is due to differences in glycosylation.

Like other LRR protiens, LRG1 has also been shown to be involved in protein-protein interactions. Serum-derived LRG1 was previously reported to bind cytochrome c [10]. We were therefore interested in determining if the differentially glycosylated isoform of LRG1 derived from human neutrophils retains the capacity to bind cytochrome c. To assess this, supernatants isolated from neutrophils stimulated with calcium ionophore (releasates) were loaded onto a cytochrome c affinity column and the retained proteins eluted with an acidic buffer. Fig 4B demonstrates clear retention of LRG1 to the cytochrome c affinity column, which is dissociated by elution with an acidic buffer. LRG1 is readily detected in the eluate after decreasing the pH of the mobile phase. These results indicate that LRG1 exocytosed from neutrophils retains affinity for cytochrome c, despite the difference in its glycosylation state.

### LRG1 modulates TGFβ1 signaling in cultured bone marrow cells

**Fig 5:**
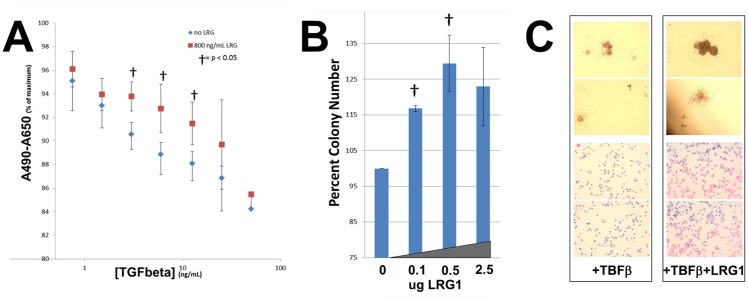
LRG1 modifies the effects of TGFβ on myeloid and hematopoietic progenitor cell growth. **(A)** HL-60 cell proliferation in the presence of increasing concentrations of TGFβ alone (blue diamonds) or TGFβ plus LRG1 (800 ng/mL, red squares) was assessed using the XTT assay. A dose-response reduction in cell proliferation as measured by the decreasing A490-A650 is observed with increasing concentrations of TGFβ that is mitigated by the addition of LRG1. The Student's t-test was used to determine the significance of the differences between control and LRG1 treated samples († p < 0.05) (n = 5). **(B)** Purified bone marrow-derived CD34+ cells were cultured in serum-free semi-solid media in the presence of TGFβ (10 ng/mL) with or without LRG1 at the indicated concentrations, and colony numbers scored. An increase in colony numbers relative to TGFβ1 treatment alone (0) is seen with increasing concentrations of LRG1. The Student's t-test was used to determine the significance of the differences between control and LRG1 treated samples († p < 0.05) (n = 3). **(C)** The effects of TGFβ alone (left panel) and TGFβ plus LRG1 (right panel) on colony formation and colony composition were analyzed. *Upper two images in each panel*. Representative light microscopy images of methocult plates on which bone marrow-derived CD34+ cells were cultured in the presence of TGFβ alone (left) or TGFβ with LRG1 (right). *Lower two images in each panel*. Wright-Giemsa staining of cells plucked from individual colonies from the methocult plates in the upper images is shown. Cells were grown as described in (B) in the presence of TGFβ (10 ng/mL) with or without LRG1 (0.625 ug/mL). LRG1 mitigates the inhibitory effect of TGFβ on the growth and size of colony-forming units-granulocyte monocyte (CFU-GM). The data shown are representative of five independent experiments, each with a different normal donor.

TGFβ1 has been shown to inhibit proliferation of HL-60 cells and the growth of hematopoietic progenitor cells [21, 22]. Recently, LRG1 was reported to modulate TGFβ1 signaling in endothelial cells by binding to TGFβ-accessory receptors [15]. We were therefore interested in determining whether LRG1 also modifies the effects of TGFβ on hematopoietic progenitor cell growth. We initially examined the effects of LRG1 and increasing concentrations of TGFβ1 on HL-60 cell proliferation using the XTT assay (Fig 5A). As expected, proliferation of HL-60 cells decreased in the presence of increasing concentrations of TGFβ1. Culture of HL-60 cells in the presence of added LRG1 alone had no appreciable effect on cell proliferation. Notably, when LRG1 was added along with TGFβ1, the previously observed decrease in proliferation of HL-60 cells induced by TGFβ1 was mitigated (Fig 5A). To determine whether LRG1 has any effect on TGFβ1 signaling in primary human hematopoietic progenitor cells, colony assays of bone marrow-derived CD34+ selected hematopoietic progenitor cells from five different healthy donors were performed using serum-free semi-solid media (SF Methocult H4436, Stemcell) to which TGFβ1 was added with or without LRG1. After 14 days in culture, total colony numbers in each plate were counted; in each individual experiment colony numbers were normalized to the total number of colonies on the TGFβ1 treated plate. The addition of TGFβ1 inhibited the growth of hematopoietic progenitor cells as indicated by a decrease in total colony number, in agreement with previously reported data [22], while addition of LRG1 alone had no effect (data not shown). When LRG1 was added to culture media containing TGFβ1, the inhibitory effect of TGFβ1 on colony formation was reversed, and total colony numbers increased, as evidenced by the observed increase in colony numbers on the LRG1 treated plates relative to the TGFβ1 only treated plates (Fig 5B). Similarly, the observed inhibitory effect of TGFβ1 on colony growth was reversed by LRG1 treatment, as seen by both larger colony size (Fig 5C, upper panels) and increased cell number in each colony (Fig 5C, lower panel). The antagonistic effect of LRG1 on TGFβ1-induced growth inhibition was most marked for colony forming units-granulocyte/monocyte (CFU-GM) as demonstrated by light microscopic examination of Wright-Giemsa stained slides of cells plucked from individual colonies (Fig 5C, lower panels), which was confirmed in three independent experiments.

## Discussion

Previous work by our laboratory to identify differentially regulated genes expressed during neutrophilic granulocyte differentiation led to identification of the genes for murine and human LRG1 [9]. Localization of the human gene for LRG1 by our group to a region on chromosome 19 where the genes for multiple neutrophil granule proteins also map prompted us to postulate that LRG1 might be a neutrophil granule protein. Using murine myeloid cell lines transfected with a tagged LRG1 cDNA construct, LRG1 was found to be packaged into the primary granules of differentiating cells [23]. A drawback of these studies was the use of an overexpression system, which can lead to inappropriate targeting of proteins to subcellular compartments that differ from their native counterparts in primary cells. We therefore sought to characterize LRG1 in its native state in unmanipulated primary cells. Additionally, since our earlier studies were done in murine cells, we were interested in examining LRG1 in human cells. In the present study, we provide the first evidence that LRG1 is a genuine human neutrophil granule protein that is released upon neutrophil activation, and exhibits distinct physical and biological properties.

We initially examined LRG1 protein expression in HL-60 cells induced to undergo neutrophilic differentiation in response to ATRA. LRG1 protein levels increased during differentiation toward the neutrophil stage, corroborating our earlier finding in murine cells demonstrating that LRG1 transcription is upregulated during granulopoiesis [9]. Using immunofluorescence microscopy, we could show that LRG1 is, indeed, packaged into neutrophil granules and co-localizes with LF to the secondary granule compartment. Localization of LRG1 to secondary granules was also confirmed using standard methods for density gradient separation of subcellular fractions from neutrophils to identify the contents of individual neutrophil granule subtypes [17].

Protein packaging into the various neutrophil granule subsets has previously been shown to be regulated by the timing of expression of proteins during granulopoiesis; thus, proteins expressed early in differentiation are packaged into primary granules, while proteins expressed during more terminal stages of maturation are targeted to tertiary granules [24]. Notably, in our earlier studies in which LRG1 was transfected into murine myeloid cell lines and overexpressed, it was found to localize to the MPO-containing primary granules [17]. In this model system, ectopic expression of LRG1 was driven by a constitutive CMV promoter [23]. It is not surprising then that constitutively expressed LRG1 was found to co-localize with MPO, since LRG1 expression occurred concomitant with MPO expression. In the present study, although native LRG1 protein is predominantly found in the secondary granules of human neutrophils, it is also present to a lesser extent in the gelatinase-containing granules. Co-localization to more than one neutrophil granule compartment is not unique to LRG1, and has previously been reported for haptoglobin and CRISP3 [20, 25].

We also demonstrate that like other neutrophil granule proteins, LRG1 is released from neutrophils following stimulation with known secretagogues [19, 26]. The pattern of secretion of LRG1 is consistent with release from the secondary granule compartment. In addition, we show that neutrophil-derived LRG1 undergoes further post-translational modifications to account for its higher molecular weight compared to serum-derived LRG1.

Aside from the canonical role of neutrophils in innate immunity as first responders in pathogen elimination, other functions have recently been ascribed to neutrophils in both innate and adaptive immunity [27, 28]. Several neutrophil granule proteins have been shown to exhibit anti-microbial properties as well as the capacity to regulate the function of other cells of the immune system. A number of neutrophil granule proteins have been shown to activate monocytes and macrophages [29].

Recently, LRG1 was reported to modify TGFβ1 signaling in endothelial cells via protein:protein interactions [15]. TGFβ1 is a ubiquitous signaling molecule that elicits responses in a variety of cell types [30, 31]. TGFβ isoforms have been shown to inhibit myelopoiesis and proliferation of hematopoietic progenitors [32]. Our data demonstrating that LRG1 alters TGFβ1 signaling in myeloid and hematopoietic progenitor cells suggests a novel role for LRG1 as a potential modulator of the marrow microenvironment and at sites of inflammation where neutrophils accumulate. The antagonistic effect of LRG1 on TGFβ1 signaling in myeloid cells is opposite to that previously reported in endothelial cells; this observed dissimilarity may relate to inherent differences in receptor and signaling molecule expression in the different cell types. Future studies with LRG1 will help to further elucidate its role, particularly *in vivo*, in modulating the pleiotropic effects of TGFβ1.

## Supporting Information

S1 Fig (TIF)
