# Supplementary figures and images for: Leucine Rich α-2 Glycoprotein: A Novel Neutrophil Granule Protein and Modulator of Myelopoiesis
Source: PLoS One. 2017 Jan 12;12(1):e0170261. doi: 10.1371/journal.pone.0170261 (PMC5233425; doi:10.1371/journal.pone.0170261)

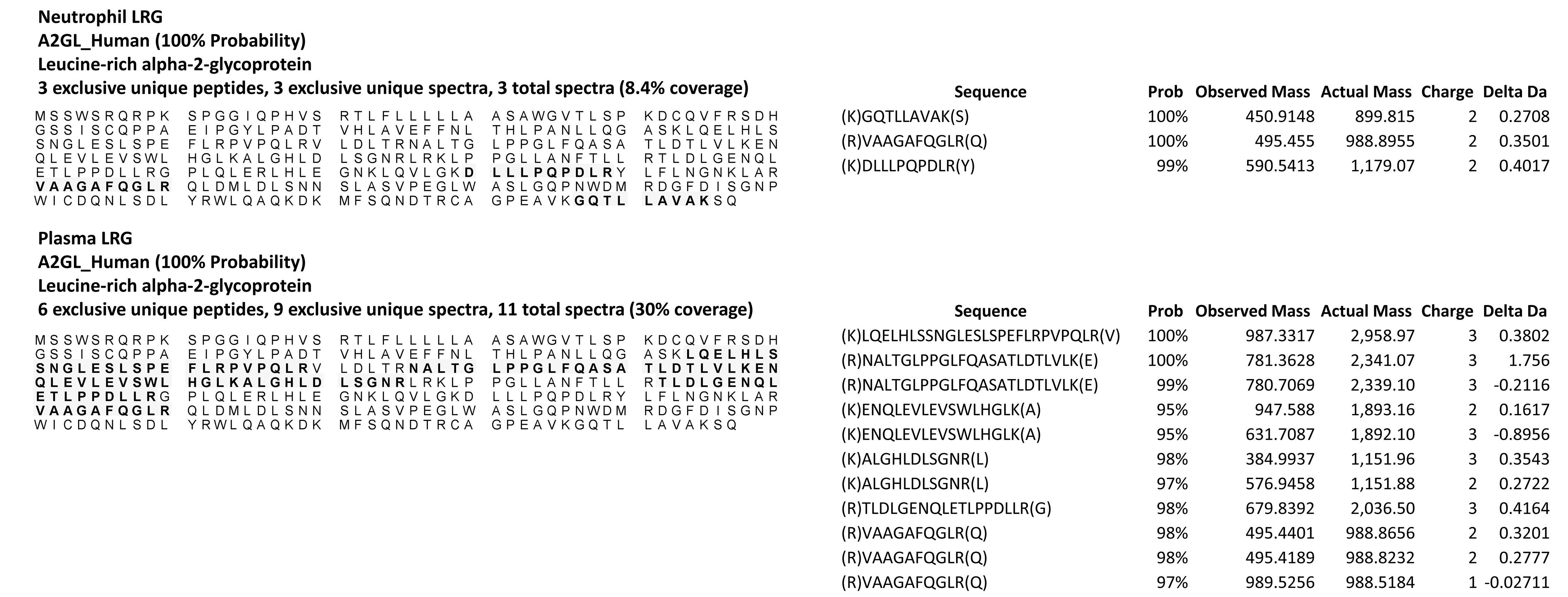

Supplement: S1 Fig — (TIF) [file pone.0170261.s001.tif]
